# Gut microbiome and reproductive endocrine diseases: a Mendelian randomization study

**DOI:** 10.3389/fendo.2023.1164186

**Published:** 2023-08-04

**Authors:** Ye Liang, Weihong Zeng, Tao Hou, Haikun Yang, Boming Wu, Ru Pan, Lishan Huang

**Affiliations:** Department of Gynecology, Meizhou People’s Hospital, Meizhou, Guangdong, China

**Keywords:** Mendelian randomization, polycystic ovary syndrome, gut microbiome, endometriosis, female infertility

## Abstract

**Background:**

Observation studies have confirmed the association between the gut microbiome and reproductive endocrine diseases (REDs), namely, polycystic ovary syndrome (PCOS), endometriosis, and female infertility. However, their association has never been confirmed by a two-sample Mendelian randomization (MR) analysis.

**Methods:**

We conducted a two-sample MR analysis to evaluate the relationship between the gut microbiome and the three aforementioned REDs. In order to get more comprehensive results, two different thresholds were adopted to select instrumental variables (IVs): one was a locus-wide significance threshold (*P <*1.0×10^–5^) and the other was a genome-wide significance level (*P*< 5×10^-8^). Summary-level statistics for the gut microbiome and REDs were collected from public databases. Inverse-variance weighted (IVW) was the main method used to estimate causality, and sensitivity analyses were conducted to validate the MR results.

**Results:**

At the locus-wide significance level, we identified that the genera Streptococcus (OR=1.52, 95%CI: 1.13-2.06, *P*=0.006) and RuminococcaceaeUCG005 (OR=1.39, 95%CI: 1.04-1.86, *P*=0.028) were associated with a high risk of PCOS, while Sellimonas (OR= 0.69, 95%CI: 0.58-0.83, *P*=0.0001) and RuminococcaceaeUCG011(OR=0.76, 95%CI: 0.60-0.95, *P*=0.017) were linked to a low PCOS risk. The genus Coprococcus2 (OR=1.20, 95%CI: 1.01-1.43, *P*=0.039) was correlated with an increased risk of female infertility, while Ruminococcus torques (OR=0.69, 95%CI: 0.54-0.88, *P*=0.002) were negatively associated with the risk of female infertility. The genera Olsenella (OR= 1.11, 95%CI: 1.01-1.22, *P*=0.036), Anaerotruncus (OR= 1.25, 95%CI: 1.03-1.53, *P*=0.025), and Oscillospira (OR= 1.21, 95%CI: 1.01-1.46, *P*=0.035) were linked to a high risk of endometriosis. However, the results showed that the gut microbiome did not possess a causal link with REDs risk based on the genome-wide significance level. Sensitivity analyses further confirmed the robustness of the MR results.

**Conclusion:**

Our study provides evidence that gut microbiome is closely related with REDs. Subsequent studies should be conducted to promote microbiome-orientated therapeutic strategies for managing REDs.

## 1 Introduction

The homeostasis of sex hormones plays a significant role in the reproductive endocrine system throughout the lifetime of a female. Disturbance in sex hormones may lead to reproductive endocrine diseases (REDs) such as polycystic ovary syndrome (PCOS), endometriosis, and infertility that have bothered female people of reproductive age for many years. PCOS is one of the most prevalent endocrine and metabolic disorders in reproductive-aged female people. Female people presenting PCOS have a high prevalence of endocrine–metabolic dysfunction, including obesity, insulin resistance, hyperinsulinemia, and dyslipidemia, resulting in a significantly increased risk for mood disorders, type 2 diabetes mellitus, infertility, metabolic disorders, cardiovascular disorders, and the development of cancer (1–3). Infertility is defined as the failure to conceive after 12 months of regular unprotected sexual intercourse. The causes of infertility include male factors, female factors, and unknown factors. It has become a major public health problem affecting 8-12% of reproductive-aged couple (4). Endometriosis is a disease characterized by endometrial tissue outside the uterus, which affects 10% of reproductive-aged female people worldwide and leads to chronic painful symptoms and infertility in severe cases (5). Due to the health, economic, and social burdens caused by these diseases, it is urgent to understand the underlying mechanisms and obtain an adequate treatment for them.

Growing evidence has revealed the relationship between the gut microbiome and REDs. The gut microbiome is considered to be an endocrine organ and plays a major role in the reproductive endocrine system by affecting the fluctuation of sex hormones. The gut microbiome can affect estrogen levels by modulating the secretion of β-glucuronidase. The dysbiosis and reduction of gut microbiota diversity can decrease or increase β-glucuronidase activity and result in the fluctuation of circulating estrogens, which may lead to obesity, metabolic syndrome, cancer, endometrial hyperplasia, endometriosis, PCOS, and infertility (6, 7). The gut microbiome can also affect the level of circulating testosterone. The gut microbiota can synthesize and transform androgens by expressing the enzymes and are involved in the degradation of testosterone *via* microbial processes (8). For example, Proteobacteria can degrade androgen (9), and Clostridium scindens has a high potential to convert glucocorticoids into androgens (10). A combination of signs and symptoms of hyperandrogenism is a typical feature of PCOS. Previous studies found that the gut microbiome and its metabolites played an important role in the regulation of PCOS-associated ovarian dysfunction and insulin resistance (11). Obesity and PCOS also have a reverse effect on changing the gut microbiome composition, which may disrupt the ovarian function, damage oocyte quality, and cause chronic inflammation, hence further deteriorating fertility (12). Thus, the gut microbiome may have an impact on PCOS pathogenesis through a variety of mechanisms.

The majority of instances of female infertility can be explained in terms of ovulation disorders, uterine or cervical issues, tubal alterations, endometriosis, immune factors, and/or pelvic infections. However, approximately 30% of cases cannot be explained, and these are defined as “unexplained infertility” (13). Growing evidence has confirmed that gut microbiota dysbiosis has an indispensable impact on inflammatory conditions that affect male and female fertility (14, 15). An observational study found that female infertility showed a different bacterial richness and ratio, and an increasing level of inflammation comparing with the fertile group (15). A systematic review demonstrated that many autoantibodies, such as thyroid-related autoantibodies, anti-phospholipid antibodies, and anti-nuclear antibodies, impede the chances of a successful *in vitro* fertilization cycle (16). Therefore, we concluded that gut microbiome may have a close relationship with female infertility.

The understanding of the etiology of endometriosis is still lacking. Recently, studies have shown that the gut microbiome may be closely associated with the onset and progression of endometriosis due to its influence on the estrogen metabolism and inflammation. The increasing level of circulating estrogen derived by gut microbiome dysbiosis may stimulate the growth and cyclic bleeding of endometriotic lesions (8). Another reason is that dysbiosis in the gut microbiome disrupts the immune function, leading to the elevation of inflammatory cytokines and alteration of immune cell profiles. Over time, a chronic state of inflammation is developed to create an environment conducive to increased adhesion and angiogenesis, which may drive endometriosis onset and progression (17). The gut microbiome also contributes to the chronic pain of endometriosis by regulating microglia, astrocytes, and immune cells, and gut microbiome dysbiosis could lead to incorrect immune responses (18).

These above observations indicate there is a close link between the gut microbiome and the pathogenesis and progression of REDs; however, a Mendelian randomization (MR) analysis about their associations is still lacking. It is necessary to establish a causal relationship analysis to further understand the gut microbiome-derived mechanism and provide new insights into microbiome-orientated therapeutic strategies. Hence, we conducted a two-sample MR analysis to evaluate the relationship between gut microbiome composition and REDs. MR is an effective method to infer causality between exposures and outcomes by using genetic variations strongly associated with exposures as instrumental variables (IVs). MR can be regarded as a natural randomized controlled trial (RCT), which is not easily disturbed by confounding factors and has a high level of evidence.

## 2 Materials and methods

### 2.1 Data sources

Genome-wide association studies (GWAS) data sources for the gut microbiome and PCOS, pregnancy loss, female infertility, and endometriosis were compiled and made publicly available online (**Table 1**). Single-nucleotide polymorphisms (SNPs) associated with the composition of the human gut microbiome were selected as IVs. Ethics approval was not required, since the data used were obtained from published studies or public databases.

**Table 1:** Summary of genome-wide association studies (GWAS) datasets in our study.

Phenotype	Type of trait	Source	Ethnicity	Sample Size	No.of cases	No. of SNPs	References
Gut Microbiome	Genus	MiBioGen	Multi-ancestry	18,473	–	122,110	(19)
PCOS	Binary	Day et al.	European	113,238	10,074	9,295,102	(20)
Female Infertility	Binary	FinnGen	European	104,225	9,831	16,381,204	(21)
Endometriosis	Binary	OpenGWAS	European	77,257	8,288	16,377,306	(22)

"-" The group of cases were not set in the GWAS study of gut microbiome.

#### 2.1.1 Gut microbiome

GWAS summary statistics for the human gut microbiome were obtained from the MiBioGen study, which is a large-scale, multiethnic GWAS study recruiting 18, 473 individuals (24 cohorts) from various countries with 122,110 loci of variation (19). Most of the participants had European ancestry (n = 13, 266). A total of 211 taxa were categorized by five biological categories, including 9 phyla, 16 classes, 20 orders, 35 families, and 131 genera. As the genus was the smallest and most precise taxonomic level among all the category criteria, we performed subsequent analyses at the genus level only. Therefore, a total of 119 specific genera were included in the current analysis after removing 12 unknown genera out of 131 genera.

#### 2.1.2 PCOS

GWAS data for PCOS were taken from Apollo (https://doi.org/10.17863/CAM.27720), which includes 10,074 PCOS cases and 103, 164 controls of European ancestry (20). Cases were either diagnosed according to the National Institutes of Health (NIH) or Rotterdam criteria or self‐reported history of PCOS.

#### 2.1.3 Female infertility

Summary-level data for female infertility were also derived from the FinnGen consortium (104,225 female participants recruited, including 9, 831cases and 94,394 controls) (21).

#### 2.1.4 Endometriosis

The GWAS summary datasets for endometriosis were accessed through the OpenGWAS database (22). The diagnostic criterion of endometriosis based on the International Classification of Diseases 10th code and GWAS ID is finn-b-N14_ENDOMETRIOSIS, with 77, 257 female participants recruited, including 8,288 cases and 68, 969 controls.

### 2.2 Selection of IVs

In this study, several steps were conducted to select eligible SNPs as IVs from the exposure data. First, SNPs strongly associated with the gut microbiome were selected. In order to obtain more comprehensive results, two different thresholds were adopted to select IVs: (1) SNPs at the locus-wide significance threshold (*P* < 1.0×10^–5^) were selected as potential IVs; (2) SNPs at the genome-wide significance level (*P*< 5 × 10^-8^) were selected as potential IVs. Second, to ensure that IVs used for the gut microbiome were independent, we excluded SNPs that had the linkage disequilibrium (LD) effect (r^2^ < 0.001, clumping window = 10,000kb). Third, SNPs related to confounders and risk factors for outcome were removed from the analysis by using the online database “PhenoScanner” (http://www.phenoscanner.medschl.cam.ac.uk/) with the filtration of r ^2^ > 0.8 and *p* < 1 × 10^−5^. The IVs of the gut microbiome identified above were extracted from each outcome dataset. Proxy SNPs were not sought by default when specific SNPs were absent in the outcome GWAS. Palindromic SNPs were also excluded. Afterwards, the exposure data and the outcome data were harmonized, which means that the effect of the SNP on the exposure was reconciled with the effect on the outcome in terms of the same allele. The strength of the included IVs was assessed with the F-statistics and R^2^. R^2^ reflects the degree to which the IV explains the exposure and is calculated as formula R^2 =^ 2 × EAF × (1 − EAF) ×β^2^/[2 × EAF × (1 − EAF) × β^2 +^ 2 × EAF × (1 − EAF)×N × se^2^] (EAF: effect allele frequency, se: the standard error for effect size, β: the effect size, N: the sample size) (23).The F-statistic was calculated by the formula F = R2 × (N − 2)/(1 − R2) (N:the sample size), where weak instrument bias is relatively low with an F-statistic over 10 (24).

### 2.3 MR analysis

An MR analysis was performed to determine if there is a causal relationship between the gut microbiome and the risk of REDs by using inverse-variance weighted (IVW) as the main method and other methods too, including MR-Egger, weighted median, and weighted mode. IVW was conducted to estimate the causality of each SNP with the assumption of no pleiotropy in these SNPs (25). Comparing with IVW, the MR-Egger not only allows the presence of pleiotropy in > 50% of IVs, but also detects horizontal pleiotropy in term of its intercept with a y-axis (26, 27). There is horizontal pleiotropy when the intercept is not zero. Point estimates from IVW MR are close to that of the MR-Egger when the intercept is close to zero. The weighted median was performed when the presence of pleiotropy was < 50% in IVs (28). The weighted mode method had less power to detect causal effects than the IVW and weighted median methods, but it was larger than that of MR-Egger and presented less bias than the above methods (29).

### 2.4 Sensitivity analysis

Cochran’s Q test was used to access heterogeneity (30). The MR-Egger regression test was performed to detect pleiotropy. There was horizontal pleiotropy when the intercept was not zero (26). MR-PRESSO was performed to reduce horizontal pleiotropy by detecting and removing final outliers (31). The leave-one-out sensitivity analysis was implemented to validate the robustness of the results by removing a single SNP each time.

A reverse MR analysis was not performed due to the lack of SNPs (related to REDs). All statistical analyses were performed using the package “TwoSampleMR” and “MR-PRESSO” in the R software (Version 4.2.0). Considering multiple-testing correction, FDR correction (Q-value) was performed using the Benjamini–Hochberg method.

## 3 Results

### 3.1 Instrumental variables selection

Initially, a total of 7098 SNPs categorized by 119 genera were extracted under the threshold of the locus-wide statistical significance (*P*< 1× 10^-5^). There was no genus containing only one SNP for each outcome dataset. In the present study, the F-statistic of IVs were all over 10, indicating no evidence of weak instrument bias. Detailed information including effect allele, other allele, Beta, SE, *P*-value, and F-statistics in IVs is shown in **Supplementary Tables 1A–C**. We evaluated the causal effect of each genus on the outcome data.

A total of 396 SNPs were extracted under the threshold of genome-wide statistical significance (*P*<5×10^-8^). After a series of quality control steps, a total of 12 independent SNPs were identified as IVs for PCOS, 11 independent SNPs for female infertility, and 11 independent SNPs for endometriosis. There was no weak instrument bias, as the F-statistics of IVs were all greater than 10. Detailed information including effect allele, other allele, Beta, SE, and *P*-value on IVs is shown in **Supplementary Table 4A**. Due to the limited number of IVs that met the requirements and each IV representing different genera, we took them as a whole to identify the gut microbiome to estimate its causal effect on outcome data.

### 3.2 Two-sample MR analysis(locus-wide significance level, *P<*1×10^-5^)

We conducted an MR analysis to evaluate the causal relationship between each genus and PCOS, female infertility, and endometriosis. The comprehensive results are shown in **Supplementary Tables 2A–C**. Streptococcus, Sellimonas, RuminococcaceaeUCG011, and RuminococcaceaeUCG005 were found to be associated with PCOS when evaluated by IVW. The IVW estimate suggested that the genera Streptococcus (OR:1.52, 95% confidence interval (CI):1.13-2.06, *P*=0.006) and RuminococcaceaeUCG005(OR:1.39, 95%CI:1.04-1.86, *P*=0.028) are positively associated with PCOS risk, while Sellimonas (OR:0.69,95%CI: 0.58-0.83, *P*=0.0001) and Ruminococcaceae UCG011(OR:0.76, 95%CI: 0.60-0.95, *P*=0.017) are negatively associated with PCOS risk (**Table 2**). However, only Sellimonas was still significant after FDR correction (Q-value= 0.015) (**Supplementary Table 3**). Ruminococcus torques and Coprococcus2 were found to be associated with female infertility when evaluated by IVW. The IVW estimate suggests that the genus Coprococcus2 (OR:1.20, 95%CI:1.01-1.43, *P*=0.039) is associated with an increased risk of female infertility, while Ruminococcus torques (OR:0.69, 95%CI:0.54-0.88, *P*=0.002) is associated with a decreased risk of female infertility (**Table 2**). However, no causal association of these genera with female infertility was supported after FDR correction (**Supplementary Table 3**). Olsenella, Anaerotruncus, and Oscillospira were found to be associated with endometriosis when evaluated by IVW. The IVW estimate suggested that the genera Olsenella (OR:1.11, 95%CI: 1.01-1.22, *P*=0.036), Anaerotruncus (OR:1.25, 95%CI: 1.03-1.53, *P*=0.025), and Oscillospira (OR:1.21,95%CI: 1.01-1.46, *P*=0.035) are associated with an increased risk of endometriosis (**Table 2**), while these associations were no longer significant after FDR correction (**Supplementary Table 3**).

**Table 2:** Causal links between gut microbiota and REDs in the MR analysis. (*P*<1×10-5).

Bacterial genera (exposure)	Outcomes	nSNPs	Methods	OR(95%CI)	Beta	Se	*P* value	Horizontal pleiotropy			Heterogeneity		MR-PRESSO
								Egger intercept	SE	*P* value	Cochran’s Q	*P* value	*P* value
Streptococcus	PCOS	15	MR Egger	2.36 (0.80-6.96)	0.86	0.55	0.145	-0.03	0.04	0.43	6.61	0.92	0.97
			IVW	1.52 (1.13-2.06)	0.42	0.15	0.006				7.28	0.92	
			Weighted median	1.62 (1.07-2.46)	0.48	0.21	0.023						
			Weighted mode	1.70 (0.89-3.25)	0.53	0.34	0.128						
Sellimonas	PCOS	9	MR Egger	1.14(0.38-3.43)	0.13	0.56	0.826	-0.07	0.08	0.41	3.93	0.79	0.50
			IVW	0.69(0.58-0.83)	-0.36	0.09	0.0001				4.72	0.79	
			Weighted median	0.69(0.54-0.88)	-0.37	0.13	0.003						
			Weighted mode	0.70(0.47-1.05)	-0.35	0.21	0.123						
RuminococcaceaeUCG011	PCOS	8	MR Egger	0.32 (0.11-0.91)	-1.13	0.53	0.077	0.11	0.07	0.15	6.51	0.37	0.275
			IVW	0.76 (0.60-0.95)	-0.28	0.12	0.017				9.40	0.23	
			Weighted median	0.75 (0.57-0.95)	-0.28	0.14	0.049						
			Weighted mode	0.61 (0.37-1.02)	0.19	0.42	0.117						
RuminococcaceaeUCG005	PCOS	14	MR Egger	1.20(0.53-2.72)	0.19	0.42	0.664	0.01	0.03	0.72	11.91	0.45	0.35
			IVW	1.39 (1.04-1.86)	0.33	0.15	0.028				12.04	0.52	
			Weighted median	1.33 (0.89-1.89)	0.28	0.20	0.164						
			Weighted mode	1.23 (0.65-2.31)	0.20	0.32	0.541						
Ruminococcustorques	Female infertily	7	MR Egger	1.15(0.56-2.35)	0.14	0.36	0.716	-0.04	0.02	0.19	1.99	0.85	0.21
			IVW	0.69 (0.54-0.88)	-0.38	0.12	0.002				4.27	0.64	
			Weighted median	0.80 (0.59-1.10)	-0.22	0.16	0.168						
			Weighted mode	0.86 (0.54-1.36)	-0.15	0.24	0.542						
Coprococcus2	Female infertily	8	MR Egger	1.09 (0.28-4.27)	0.09	0.70	0.903	0.01	0.05	0.90	5.01	0.54	0.07
			IVW	1.20(1.01-1.43)	0.18	0.09	0.039				5.03	0.66	
			Weighted median	1.15 (0.91-1.45)	0.14	0.12	0.222						
			Weighted mode	1.15(0.79-1.68)	0.14	0.19	0.492						
Olsenella	Endometriosis	8	MR Egger	1.03(0.75-1.40)	0.03	0.16	0.863	0.01	0.02	0.63	7.12	0.52	0.61
			IVW	1.11 (1.01-1.22)	0.10	0.05	0.036				7.37	0.60	
			Weighted median	1.11 (0.97-1.27)	0.10	0.07	0.146						
			Weighted mode	1.04 (0.85-1.26)	0.04	0.10	0.715						
Anaerotruncus	Endometriosis	13	MR Egger	0.85(0.49-1.47)	-0.17	0.28	0.566	0.03	0.02	0.17	11.47	0.41	0.329
			IVW	1.25 (1.03-1.53)	0.23	0.10	0.025				13.76	0.32	
			Weighted median	1.24 (0.97-1.60)	0.22	0.13	0.091						
			Weighted mode	1.20 (0.86-1.67)	0.18	0.17	0.299						
Oscillospira	Endometriosis		MR Egger	0.96(0.45-2.06)	-0.04	0.39	0.918	0.02	0.04	0.55	2.88	0.82	0.742
			IVW	1.21 (1.01-1.46)	0.19	0.09	0.035				3.27	0.86	
			Weighted median	1.17 (0.93-1.47)	0.15	0.12	0.188						
			Weighted mode	1.12 (0.78-1.60)	0.11	0.18	0.553						

**Figure 1:**
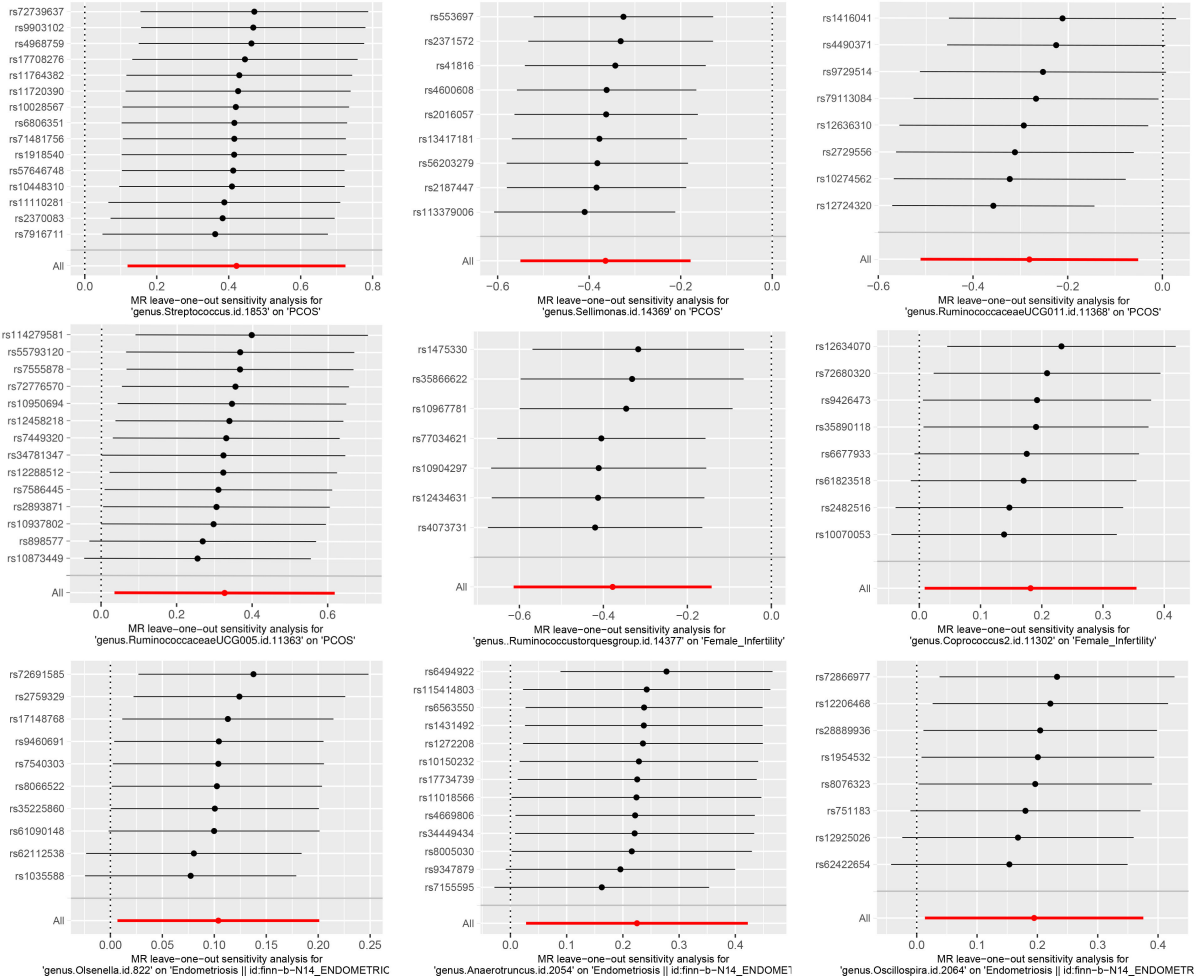
The leave-one-out sensitivity analysis assessed the associations between genera and REDs by removing a single SNP each time (P<1×10-5).

nSNPs, the number of SNPs being used as IVs; OR, odds ratio; I VW, inverse-variance weighted; PCOS, polycystic ovary syndrome; REDs, reproductive endocrine diseases;

The results of Cochran’s Q test evaluated by the IVW test and MR-Egger showed no significant heterogeneity between the gut microbiome and PCOS, female infertility, and endometriosis. There was no evidence of horizontal pleiotropy according to the results of the MR-Egger regression analysis. In addition, MR-PRESSO analysis did not find any significant outliers (**Table 2**). The leave-one-out results further validated data robustness (**Figure 1**).

### 3.3 Two-sample MR analysis(genome-wide significance level, *P*<5×10^-8^)

Considering the gut microbiome as a whole, the results of the MR analysis evaluated by IVW (OR = 1.06, 95% CI 0.87-1.30, *P* =0.58) did not show a significant causal relationship between the gut microbiome and PCOS. The other methods showed directionally consistent results (**Supplementary Table 4B**). We also could not find a causal link between the gut microbiome and female infertility (IVW: OR = 0.98, 95% CI 0.86-1.13, *P* =0.82) and endometriosis (IVW: OR = 0.96, 95% CI 0.86-1.09, *P* =0.56). Cochran’s Q statistics of the IVW test and the MR-Egger regression, respectively, showed no significant heterogeneity between gut the microbiome and PCOS and endometriosis. However, there was heterogeneity between the gut microbiome and female infertility; in this case, we applied the result of the weighted median as main MR result to evaluate the causal association between the gut microbiome and female infertility (OR =1.06, 95% CI 0.93-1.22, *P=*0.36). The MR-Egger regression results showed that there was no horizontal pleiotropy between the gut microbiome and PCOS, female infertility, and endometriosis. The MR-PRESSO analysis showed that there were no outliers in the analysis (**Supplementary Table 4B**). Moreover, the leave-one-out results further validated the data robustness (**Supplementary Figure 1**).

## 4 Discussion

In the current study, we conducted MR analyses to evaluate the potential causality between the gut microbiota and REDs. Based on the locus-wide significance level, we identified that the genera Streptococcus and RuminococcaceaeUCG005 were associated with a high risk of PCOS, while Sellimonas and RuminococcaceaeUCG011were linked to a low PCOS risk. The genus Coprococcus2 was associated with an increased risk of female infertility, while Ruminococcus torques was negatively associated with the risk of female infertility. Genus Olsenella, Anaerotruncus, and Oscillospira were linked to a high risk of endometriosis. However, the results showed that gut microbiome did not have a causal link with REDs risk based on the genome-wide statistical significance level.

There are many previous studies on the relationship between the gut microbiome and PCOS, with Ruminococcaceae among them (32, 33). RuminococcaceaeUCG005 is a member of the Ruminococcaceae family and is viewed as a harmful bacterium in high-fat diet (HFD)-fed rats, and it correlates with oxidative stress, metabolism genes, and body weight (34). Prior evidence indicated that increased oxidative stress and elevated inflammatory status contribute to the progression of PCOS (35, 36), and weight loss is an important part of PCOS treatment (37, 38), which might partly explain why RuminococcaceaeUCG005 is associated with a high risk of PCOS. Female people presenting PCOS characterized by a combination of signs and symptoms of androgen excess have a high prevalence of obesity, insulin resistance, and dyslipidemia. Streptococcus, considered “bad bacteria”, was previously shown to be associated with obesity and significantly higher in obese and PCOS adults (39–42). Streptococcus was positively correlated with insulin resistance, testosterone, and BMI (33, 43–45). It was also found to be involved with carbohydrate metabolism and positively associated with insulin, connecting peptide, lipopolysaccharide, and pro-inflammatory indicators (42, 46, 47).However, it was negatively correlated with short-chain fatty acids (SCFAs) (42). SCFAs, the microbial fermentation end-products, may help suppress the levels of pro-inflammation cytokines, reduce inflammation in the intestine, and maintain the homeostasis of the intestinal environment (48).SCFAs are associated with insulin releasing and blood glucose levels by supporting the health of beta cells in the pancreas, and they stimulate the secretion of glucagon-like peptide-1 (GLP-1) (49, 50). Additionally, SCFA acetate may help people control weight and support healthy weight maintenance in terms of regulating hormones (such as GLP-1), increasing metabolism, and inhibiting appetite (51). Research showed that acetate could protect ovarian function by supporting normal follicles growth and enhancing circulating 17-β estradiol through the inhibition of histone deacetylase in the rat model of PCOS (52). Additionally, it has been found that probiotics and SCFAs administration, as part of anti-obesity and diabetes interventions, could involve the modification of microbiota, the upregulation of GLP-1 production and related SCFAs, such as acetate, and increasing fasting fat oxidation and resting energy expenditure (53–55). Zhang et al. found that probiotics impact the gut microbiota and sex hormones of PCOS patients by significantly decreasing the levels of luteinizing hormone (LH) and LH/follicle-stimulating hormone (LH/FSH) and markedly increasing SCFAs (56). The above evidence implies probiotics or SCFAs administration can be beneficial as part of the treatment of female disorders. Sellimonas was considered a potential biomarker of gut homeostasis recovery, as studies found that Sellimonas intestinalis was increased in patients with colorectal cancer who recovered their intestinal homeostasis following dysbiosis caused by radical surgery combined with chemotherapy (57)and in patients with liver cirrhosis who underwent therapeutic splenectomy (58). Studies on the association between Sellimonas and PCOS are limited. We speculated Sellimonas may contribute to the recovery of intestinal homeostasis in patients with PCOS. RuminococcaceaeUCG011 was negatively correlated with the serum and hepatic lipid profiles and was significantly increased after hypoglycemic and hypolipidemic intervention in type 2 diabetic mice (59). As Ruminococcaceae UCG011 was shown to be inversely correlated with the serum levels of triglyceride (TG), cholesterol (TC), low-density lipoprotein cholesterol (LDL-C), and the hepatic levels of TC, TG, non-esterified fatty acids (NEFA), and bile acids (BAs) (59), it may show a protective effect in PCOS, as in other metabolic diseases, which is consistent with our findings.

A great deal of evidence supports the role of the gut microbiome in female infertility. Coprococcus2, associated with an increased risk of female infertility in our study, was previously found to be the characteristic genus of obese patients with PCOS (60, 61). Coprococcus2 was also found to be indirectly associated with chronic low-grade systemic inflammation induced by diets and ectopic fat in the Multiethnic Cohort-Adiposity Phenotype Study (62). Increasing evidence suggests that infertility is related to chronic low-grade inflammation characterized by increased inflammatory markers, such as C-reactive protein (CRP), IL-18, TNF-α, and IL-6 (63, 64). Diets with a marked anti-inflammatory signature have been proposed in the nutritional management of infertile patients (65). For the first time, our finding has implied the relationship between Coprococcus2 and female infertility. This may provide new insights into improving fertility through diets that could decrease the abundance of Coprococcus2. Ruminococcus torques, negatively associated with the risk of female infertility in our study, was found to be negatively correlated with pre-pregnancy body weight in a previous study (66). Several studies have shown both underweight (BMI < 19 kg/m2) and overweight (BMI 25–29.9 kg/m2) affect infertility (67, 68). A cohort study with 9232 participants from Denmark found that overweight and obese mothers with a body mass index (BMI) >25 kg/m2 may harm the fertility of fetuses, and especially, sons born to overweight mothers have higher odds of infertility. However, the study did not find an association between maternal overweight and infertility in daughters (69). Tang et al. found that being underweight with BMI < 18.5 kg/m2 is linked to reduced implantation rates, clinical pregnancy rates, and ongoing pregnancy. Rates of miscarriage were markedly increased in the overweight group relative to the normal weight group (70). We speculated that Ruminococcus torques may affect fertility through its impact on pre-pregnancy body weight. Wen at el. found that Ruminococcus torques was generated after a high-cellulose diet in a mouse model of asthma. They speculated Ruminococcus torques is closely correlated with lipid metabolism *in vivo* (71).Wang et al. discovered that the abundance of Ruminococcus torques was relatively higher after mitigation treatment of colonic inflammation in colitis. Previous studies have revealed the protective role of Ruminococcus torques in other diseases, but further studies need to reveal their protective role in female infertility.

In previous studies, many researchers have revealed the relationship between the gut microbiome and endometriosis. As we know, endometriosis development is influenced by estrogen metabolism and inflammation. The abundance of Olsenella has been found to be associated with the exacerbation of inflammation. The low-abundance and pathogenic bacterium Olsenella proliferates when inflammatory hyperthermia causes host gut microbiota disorders, which further exacerbates the inflammatory response (72), whereas the downregulation of Olsenella can reduce the possibility of inflammation in the rumen epithelium and the organism (73). The abundance of Olsenella is positively correlated with the IL-10 levels (74). IL-10 family proteins, as the main members of Th2 anti-inflammatory cytokines, have been considered a critical factor for the development of endometriosis. Accumulating evidence has demonstrated that IL-10 is sharply increased in the ectopic endometrium and peritoneal fluid of female people with endometriosis, particularly in cases of advanced endometriosis (75–78). Previous research demonstrated that IL-10 from infiltrated plasmacytoid dendritic cells may suppress immunity against endometrial implants to contribute to the development of endometriosis (78) and promote angiogenesis in the early stage of endometriosis (79). Therefore, Olsenella may be involved in the development of endometriosis by modulating the level of IL-10. Moreover, N-acetylserotonin (NAS), an endogenous metabolite, was significantly negatively correlated with Olsenella (72). It has been proposed that the NAS/melatonin ratio is linked to endometriosis pathophysiology. Endometriosis, as an estrogen-dependent condition, is usually mitigated by lowering the estrogen effects. Melatonin inhibits the ERα (80), which modulates the stage transition in endometriosis (81), suggesting that melatonin inhibits ERα-driven pathophysiology in endometriosis. Endometriosis risk is also correlated with CYP1B1 SNPs (82), which increases the backward conversion of melatonin to NAS (83). Further analysis is needed to evaluate the relationships between the NAS/melatonin ratio, Olsenella, and endometriosis. The second genus we found to have a positive association with endometriosis is Oscillospira. Oscillospira was found to associated with adiposity and metabolic dysfunction and gut inflammation and serum triglycerides in mice and humans, respectively (84).Chen et al. discovered that Oscillospira is closely related to human health, because its abundance is positively correlated with high-density lipoprotein, microbial diversity, and sleep time and is inversely correlated with blood pressure, fasting blood glucose, uric acid, triglyceride, and Bristol stool type (85). Jae-Kwon Jo found that Oscillospira was significantly higher in the HFD mice than that those in the control group, producing SCFAs such as acetate, propionate, and butyrate (86). SCFAs butyrate supplement was found to alleviate the symptoms caused by low estrogen, such as excessive osteoclastogenesis and bone loss in estrogen-deficient mice due to ovariectomy (OVX) (87, 88).The association between Oscillospira and host health, SCFAs production, and the estrogen level needs further comprehensive exploration. We speculate that Oscillospira may be involved in endometriosis etiology when gut dysbiosis and estrogen imbalance occur. Anaerotruncus was the last genus we found to positively relate to endometriosis, and it was found to associated with inflammation and obesity in previous studies (89). Li et al. confirmed that the relative abundance of Anaerotruncus decreased when anti-inflammation treatment reduced psoriasis-like inflammation in mice (90). Kong et al. found that Anaerotruncus, as a conditional pathogenic bacterium, increased in a mouse model fed by HFD and high-sucrose diets (HCDs) (91). Anaerotruncus has been positively associated with glucose intolerance and gut permeability and is involved in the pathogenesis of diabetes (92). However, the studies about the mechanism of Anaerotruncus in endometriosis etiology still lack.

Our study has several advantages. To our knowledge, this is the first MR analysis to evaluate the causal relationship between the gut microbiome and REDs. The MR design has the advantage of preventing disturbance from residual confounding and might be more convincing than observational studies. However, as the exact biological function of many genetic variants is still unknown, we cannot completely avoid the impact of horizontal pleiotropy. Thus, the results should be interpreted with caution. Moreover, we analyzed the causal effect of each taxon on REDs primarily from the genus level. This provides new insights for understanding the gut microbiome-derived mechanisms and microbiome-orientated therapeutic strategies. Several limitations should be mentioned. First, our study was unable to count the participants overlapping between the exposure and outcome GWAS, which may lead to an overestimation of the results. We were also unable to identify a reverse causal relationship between them due to the lack of an adequate number of IVs for REDs. Second, our study used gut microbiome data, including multiethnic male and female participants, whereas studies about REDs were conducted on female Europeans, which may have also influenced our results. However, we were unable to avoid this bias by conducting the sex or race subgroup analysis due to the lack of demographic data in the original research. Third, the sample size of each genus from the gut microbiome GWAS was relatively small compared with that for the REDs. Therefore, not enough IVs were identified for certain bacterial features at the genus level. Fourth, multiple statistical corrections are too rigorous and conservative, which may neglect potential genera that have a causal relationship with REDs. Therefore, we did not take the multiple testing results into account. Future studies need to plan to address these limitations.

In conclusion, we comprehensively evaluated the potential association between the gut microbiome and REDs. These strains may provide candidate biomarkers and new insights into the treatment for subsequent studies.

## Data availability statement

The original contributions presented in the study are included in the article/**Supplementary Material**. Further inquiries can be directed to the corresponding author.

## Author contributions

YL and LH designed the study. WZ, BW, and TH performed data analysis. YL, RP, HY, and LH structured the manuscript and contributed to the tables, figures, and text editing. All authors contributed to the final manuscript and approved the submitted version.
