# Supplementary figures and images for: Gut microbiome and reproductive endocrine diseases: a Mendelian randomization study
Source: Front Endocrinol (Lausanne). 2023 Aug 4;14:1164186. doi: 10.3389/fendo.2023.1164186 (PMC10436605; doi:10.3389/fendo.2023.1164186)

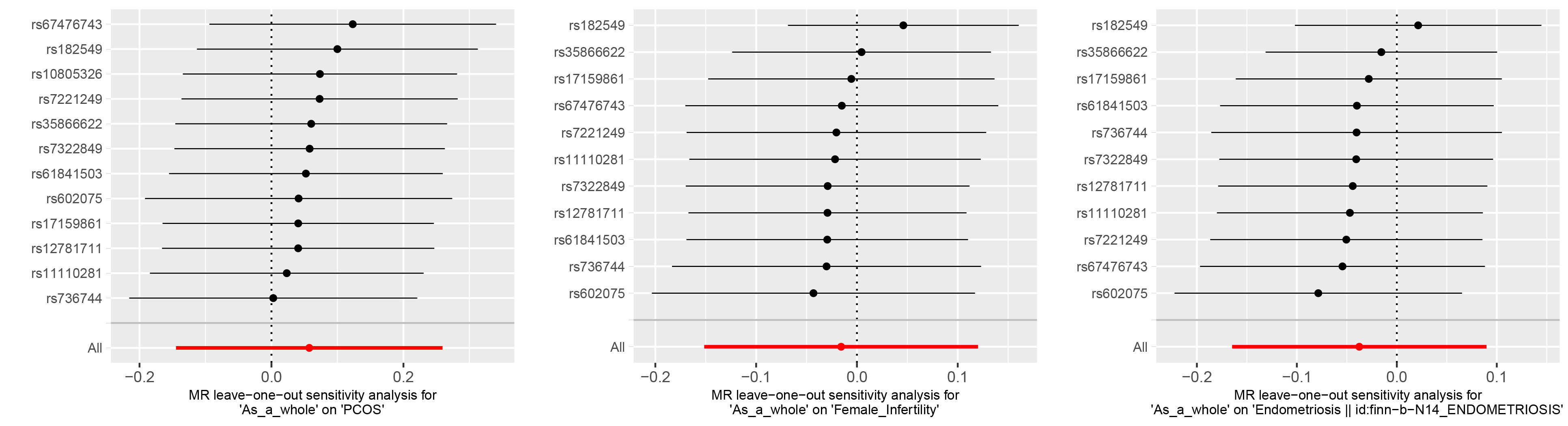

Supplement: Supplementary file 1 [file Image_1.tif]
